# Genomic Organization of Repetitive DNA in Woodpeckers (Aves, Piciformes): Implications for Karyotype and ZW Sex Chromosome Differentiation

**DOI:** 10.1371/journal.pone.0169987

**Published:** 2017-01-12

**Authors:** Thays Duarte de Oliveira, Rafael Kretschmer, Natasha Avila Bertocchi, Tiago Marafiga Degrandi, Edivaldo Herculano Corrêa de Oliveira, Marcelo de Bello Cioffi, Analía del Valle Garnero, Ricardo José Gunski

**Affiliations:** 1 Programa de Pós-graduação em Ciências Biológicas, Universidade Federal do Pampa, São Gabriel, Rio Grande do Sul, Brazil; 2 Programa de Pós-graduação em Genética e Biologia Molecular, Universidade Federal do Rio Grande do Sul, Porto Alegre, Brazil; 3 Programa de Pós-graduação em Genética, Universidade Federal do Paraná, Curitiba, Paraná, Brazil; 4 Laboratório de Cultura de Tecidos e Citogenética, SAMAM, Instituto Evandro Chagas, Ananindeua, Pará, Brazil; 5 Instituto de Ciências Exatas e Naturais, Universidade Federal do Pará, Belém, Pará, Brazil; 6 Departamento de Genética e Evolução, Universidade Federal de São Carlos, São Carlos, São Paulo, São Paulo, Brazil; Tulane University Health Sciences Center, UNITED STATES

## Abstract

Birds are characterized by a low proportion of repetitive DNA in their genome when compared to other vertebrates. Among birds, species belonging to Piciformes order, such as woodpeckers, show a relatively higher amount of these sequences. The aim of this study was to analyze the distribution of different classes of repetitive DNA—including microsatellites, telomere sequences and 18S rDNA—in the karyotype of three Picidae species (Aves, Piciformes)—*Colaptes melanochloros* (2n = 84), *Colaptes campestris* (2n = 84) and *Melanerpes candidus* (2n = 64)–by means of fluorescence *in situ* hybridization. Clusters of 18S rDNA were found in one microchromosome pair in each of the three species, coinciding to a region of (CGG)_10_ sequence accumulation. Interstitial telomeric sequences were found in some macrochromosomes pairs, indicating possible regions of fusions, which can be related to variation of diploid number in the family. Only one, from the 11 different microsatellite sequences used, did not produce any signals. Both species of genus *Colaptes* showed a similar distribution of microsatellite sequences, with some difference when compared to *M*. *candidus*. Microsatellites were found preferentially in the centromeric and telomeric regions of micro and macrochromosomes. However, some sequences produced patterns of interstitial bands in the Z chromosome, which corresponds to the largest element of the karyotype in all three species. This was not observed in the W chromosome of *Colaptes melanochloros*, which is heterochromatic in most of its length, but was not hybridized by any of the sequences used. These results highlight the importance of microsatellite sequences in differentiation of sex chromosomes, and the accumulation of these sequences is probably responsible for the enlargement of the Z chromosome.

## Introduction

It is known that the avian genome is extremely compact, corresponding to approximately one third of the typical mammalian DNA content [1]. This decrease occurred due to the loss of many genes and, in a higher proportion, DNA repetitive sequences [2]. Recently, published results of genome sequences from 48 bird species showed that their amount of repetitive sequences is much smaller than other groups of Tetrapods, corresponding to 4–10%, while in mammals the percentage of these sequences can reach up to 52% from the genome [3]. Among different classes of repetitive sequences we can find satellites, microsatellites, multigenic families, and transposable elements [3, 4]. Repetitive DNA plays an important role in genetic variation within populations, as well as in gene expression, recombination, genome structural organization, chromosomal instability and sex chromosome differentiation [5–9].

So far, the Picidae family (woodpeckers) shows the highest proportion of repetitive sequences in genomes among birds, reaching up to 22% of total DNA amount in the species *Picoides pubescens*, where the transposable element CR1 is one of the most important components [3]. In addition, karyotype analyses showed that woodpeckers have some distinctive features, such as high diploid numbers (2n), with some species possessing more than 100 chromosomes, and a large Z chromosome, the largest element of the karyotype [10–15].

In other groups of organisms, analyses of repetitive sequences have related their accumulation to the process of sex chromosomes differentiation, as in plants, reptiles and, in a greater proportion, fish [16–18]. In this regard, Matsubara and co-workers [19] observed that in *Gallus gallus* motifs (GA)_15_ and (GAG)_10_ were detected mainly in the W chromosome and, at low frequency, in the Z and autosomes. However, the organization of repetitive sequences in the genome of birds, especially their chromosomal location, is practically unknown.

The Picidae family showed a large karyotype variation in their 2n and a high amount of repetitive sequences in their genomes in comparison with other birds. Therefore, in this study we aim to characterize the chromosomal distribution of 13 classes of repetitive DNA sequences in three Picidae species, focusing on the association of these sequences with karyotype evolution and the ZW sex system differentiation.

## Material and Methods

### Animals

Ten individuals belonging to three different Picidae species (Aves, Piciformes) were used in this study: three male and one female *Colaptes campestris*, five female *Colaptes melanochloros* and one male *Melanerpes candidus* (Table 1). Animals were collected in Rio Grande do Sul State (Brazil) using mist nets (permissions SISBIO 33860–1 and 44173–1). The experiments followed protocols approved by the Ethics Committee on the Use of Animals (CEUA—Universidade Federal do Pampa, 026/2012).

**Table 1:** Specimen information and number of samples used in this study.

Species	Number of individuals and sex	City	Geographic coordinate
*C*. *campestris*	3 ♂ and 1 ♀	Dom Pedrito and São Gabriel	31°00’37.68” S; 054°36’54.29” W and 30°20’05.93” S; 054°21’47.93” W
*C*. *melanochloros*	5 ♀	Dom Pedrito and São Gabriel	31°00’37.68” S; 054°36’54.29” W and 30°20’05.93” S; 054°21’47.93” W
*M*. *candidus*	1 ♂	Porto Vera Cruz	27°42’33” S; 054°53’29” W

### Chromosome preparation

Chromosome preparations were obtained using short term cultivation of bone marrow [20] or fibroblast culture [21]. Both methods included colcemid incubation, hypotonic treatment and fixation with methanol: acetic acid (3:1).

### Classical cytogenetics

Chromosome biometry was performed using the software Micromeasure 3.3 [22]. C-banding was performed according to Fernández et al. [23], with modifications.

### Probe Preparation and Fluorescent *in situ* hybridization (FISH)

18S rDNA fragments were amplified by PCR using primers NS1 5′-GTA GTC ATA TGC TTG TCT C-3′, NS8 5′-TCC GCA GGT TCA CCT ACG GA-3′ and nuclear DNA of *Ocyurus chrysurus* (Perciformes, Lutjanidae) [24]. Afterwards, fragments were labeled with digoxigenin by nick translation (Roche) and detected with Anti-Digoxigenin-Rhodamine, following manufacturer's instructions. Telomeric probes (TTAGGG)_n_ were obtained by PCR in the absence of a DNA template, using primers (TTAGGG)_5_ and (CCCTAA)_5_ [25], labeled with biotin and detected with avidin-Cy3. Preparation of slides, hybridization and washes were performed according to Daniels and Delany [26].

Oligonucleotide probes containing microsatellite sequences (CA)_15_, (CAA)_10_, (CAC)_10_, (CAG)_10_, (CAT)_10_, (CG)_15_, (CGG)_10_, (GA)_15_, (GAA)_10_, (GAG)_10_ and (TA)_15_ were directly labeled with Cy3 during synthesis (Sigma, St. Louis, MO, USA), as described by Kubat et al. [16].

### Microscopy

At least 30 metaphase spreads per individual were analyzed to confirm the 2n, karyotype structure and FISH results. Metaphases were analyzed in an epifluorescent microscope (Imager Z2, Zeiss, Germany), and images were captured with the software Axiovision 4.8 (Zeiss, Germany). Final editing of images used Corel Photo Paint X5.

## Results

### Karyotyping and C-banding

**Fig 1:**
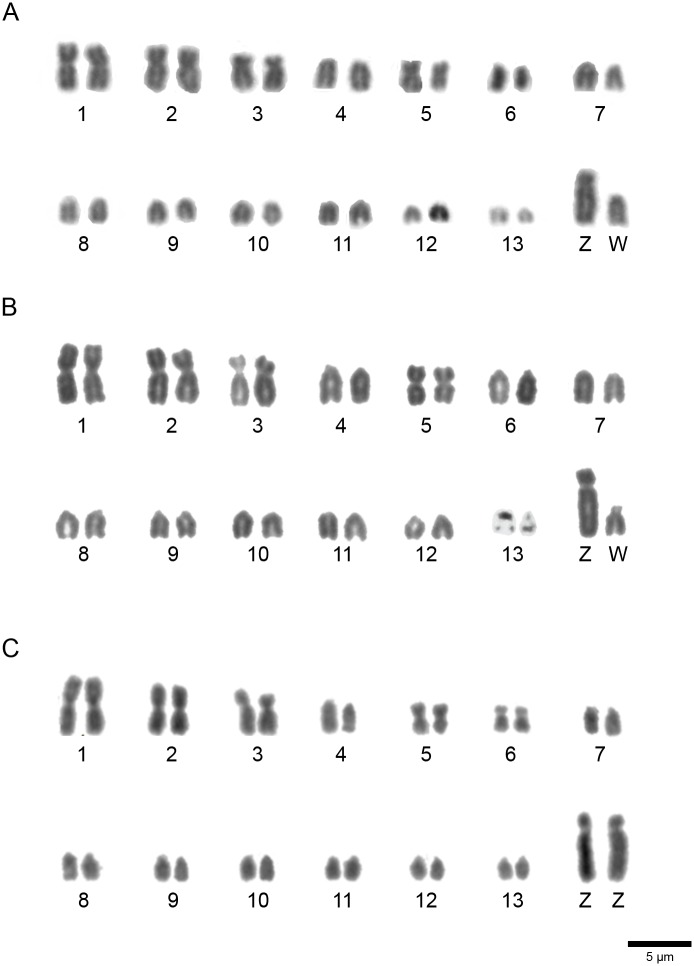
Partial karyotypes of a female *Colaptes campestris* (A), a female *Colaptes melanochloros* (B) and a male *Melanerpes candidus* (C). Bar = 5μm.

We found 2n = 84 in both species of genus *Colaptes*, while *Melanerpes candidus* showed 2n = 64 (Fig 1A, 1B and 1C). *C*. *campestris* and *C*. *melanochloros* also showed chromosomes with the same morphology, with 14 pairs of macrochromosomes, including the Z and W sex chromosomes. Chromosomal morphology of each species is described in Table 2. In all these species, the Z chromosome was acrocentric and was the largest element of the karyotype. The W was smaller, with size between the sixth and seventh pairs in *Colaptes*.

**Table 2:** Chromosomal morphology of Picidae species included in this study.

Chromosomes	*Colaptes campestris*				*Colaptes melanochloros*				*Melanerpes candidus*			
	Long arm	Short arm	CI	Morphology	Long arm	Short arm	CI	Morphology	Long arm	Short arm	CI	Morphology
**1**	1,77	1,25	41,391	M	1,72	1,18	40,690	M	1,722	1,722	50,000	M
**2**	1,59	0,96	37,647	SM	1,62	0,97	37,452	SM	1,353	1,312	49,231	M
**3**	1,6	0,53	24,883	SM	1,7	0,73	30,041	SM	1,64	0,656	28,571	SM
**4**	1,79	0,15	7,732	A	1,71	0,17	9,043	A	1,886	0,369	16,364	A
**5**	1,14	0,55	32,544	SM	1,13	0,75	39,894	SM	0,861	0,82	48,780	M
**6**	1,47	0,15	9,259	A	1,61	0,14	8,000	A	0,943	0,492	34,286	SM
**7**	1,26	0,17	11,888	A	1,38	0,14	9,211	A	1,312	0,246	15,789	A
**8**	1,37	0,09	6,164	A	1,47	0,1	6,369	A	1,23	0,328	21,053	A
**9**	1,09	0,1	8,403	A	1,17	0,1	7,874	A	1,189	0,164	12,121	A
**10**	1,15	0,13	10,156	A	1,26	0,1	7,353	A	1,107	0,328	22,857	A
**11**	1,3	0	0,000	T	1,34	0	0,000	T	1,107	0,246	18,182	A
**12**	1,1	0	0,000	T	1,16	0	0,000	T	1,025	0,287	21,875	A
**13**	1,15	0	0,000	T	0,83	0	0,000	T	1,025	0,205	16,667	A
**Z**	2,43	0,59	19,536	A	2,45	0,69	21,975	A	3,239	0,82	20,202	A
**W**	1,43	0,21	12,805	A	1,33	0,17	11,333	A	-	-	-	-

CI = Centromeric Index, M = metacentric, SM = submetacentric, A = acrocentric, T = telocentric

**Fig 2:**
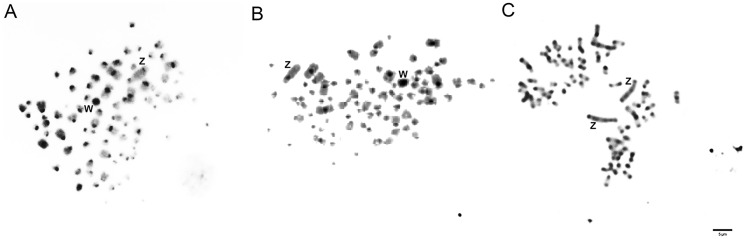
C-banded chromosomes in mitotic metaphase of *Colaptes campestris* (A), *Colaptes melanochloros* (B) and *Melanerpes candidus* (C). Chromosomes were stained with DAPI and converted to black and white with Corel^®^ photo editor. Sex chromosomes are indicated in each metaphase. Bar = 5μm.

Blocks of constitutive heterochromatin were seen in the pericentromeric region of macrochromosomes, including the Z chromosome, and some microchromosomes, in all the species analyzed (Fig 2A, 2B and 2C). The W chromosome was heterochromatic in most of its length in both *Colaptes* species (Fig 2A and 2B).

### Chromosomal mapping of repetitive elements

**Fig 3:**
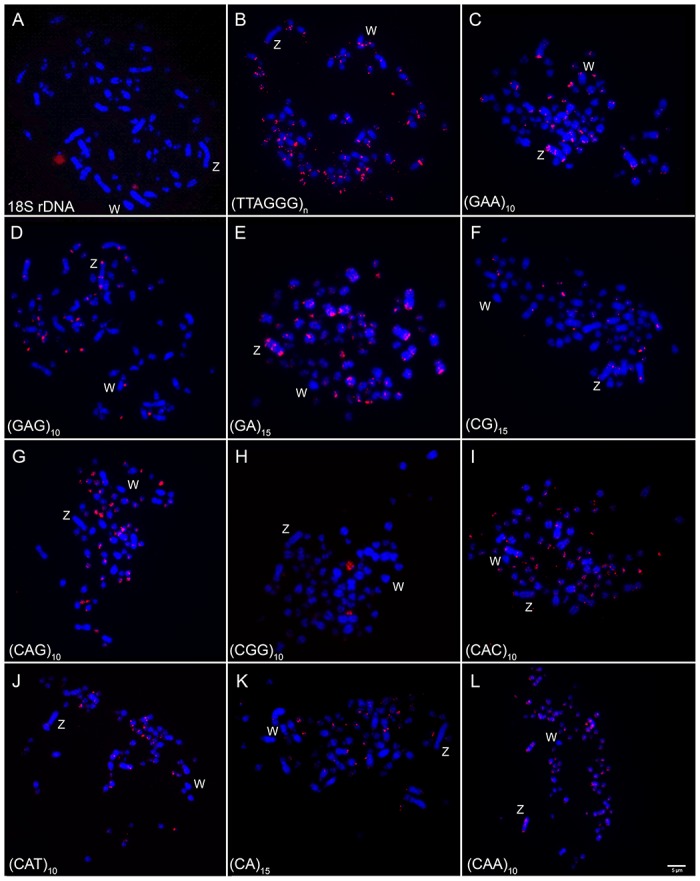
Metaphase chromosomes of a female *Colaptes melanochloros* hybridized with: 18S rDNA (A), telomeric DNA (B) and microsatellites DNA (C-G). Chromosomes were counterstained with DAPI (blue), and microsatellite probes were labeled directly during synthesis with Cy3 (red). Probes used are indicated in the lower left corner of the images. Sex chromosomes are indicated in each metaphase. Bar = 5μm.

**Fig 4:**
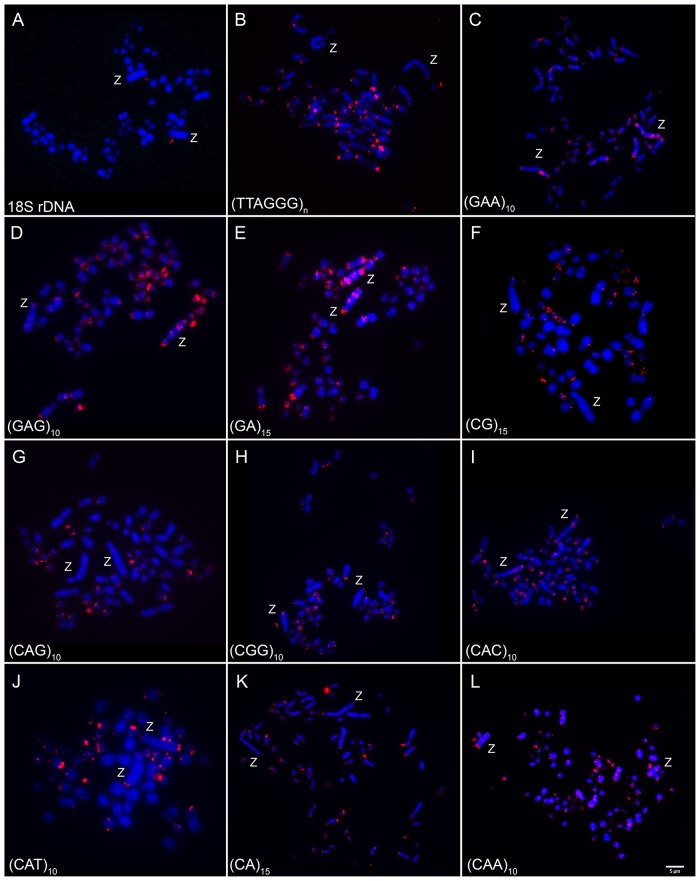
Metaphase chromosomes of a male *Melanerpes candidus* hybridized with: 18S rDNA (A), telomeric DNA (B) and microsatellites DNA (C-G). Chromosomes were counterstained with DAPI (blue) and microsatellite probes were labeled directly during synthesis with Cy3 (red). Probes used are indicated in the lower left corner of the images. Sex chromosomes are indicated in each metaphase. Bar = 5μm.

Clusters of 18S rDNA were found in only one microchromosome pair in all species analyzed (Figs 3A and 4A). According to chromosomal biometry, this chromosome correspondeds to pair 13 in both *Colaptes*, which exhibits a secondary constriction, and to pair 18 in *M*. *candidus*.

Telomeric probes produced signals only in the terminal region of all chromosomes, except for the interstitial telomere sequences (ITS) observed in the centromeric region of pairs 1–3 in both *Colaptes species* (Fig 3B) and pairs 1–2 and 5 in *M*. *candidus* (Fig 4B).

Among the 11 distinct microsatellite sequences used in this study, only (TA)_15_ did not produce signals in any of the species analyzed. The same hybridization patterns were observed in both *Colaptes* species, while *M*. *candidus* showed a distinct pattern (Table 3, Figs 3C, 3D, 3E, 3F, 3G, 3H, 3I, 3J, 3K, 3L, 4C, 4D, 4E, 4F, 4G, 4H, 4I, 4J, 4K and 4L). Accumulations of microsatellites were found mainly in centromeric and telomeric regions of the chromosomes, although some sequences produced signals in interstitial blocks.

**Table 3:** Hybridization of repetitive sequences in Picidae.

Probes	Chromosomes					
	Macrochromosomes		Microchromosomes		Z	
	COL	MCA	COL	MCA	COL	MCA
(GAA)_10_	+	+	+	+	+	+
(GAG)_10_	+	+	+	+	+	+
(GA)_15_	+	+	+	-	+	+
(CG)_15_	+	+	+	+	+	+
(CAG)_10_	+	-	+	+	-	-
(CGG)_10_	+	+	+	+	-	+
(CAC)_10_	+	+	+	+	+	+
(CAT)_10_	+	+	+	+	-	+
(CA)_15_	+	+	+	+	-	+
(CAA)_10_	+	+	+	+	+	+

(+) positive hybridization signals; (-) no hybridization signals; (COL) Genus *Colaptes*; (MCA) *Melanerpes candidus*.

**Fig 5:**
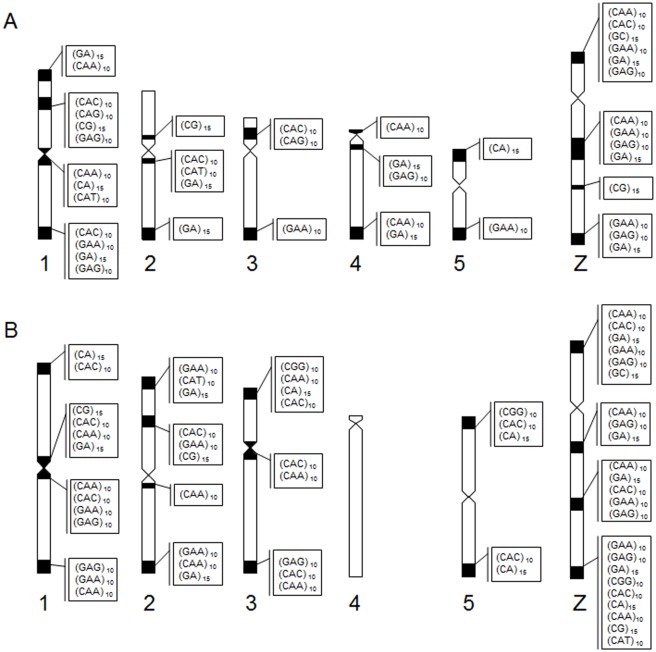
Distribution and localization of microsatellite sequences in chromosomes 1–5 and Z in *Colaptes* (A) and *M*. *candidus* (B).

Macrochromosomes showed a preferential accumulation of microsatellites in centromeric and telomeric regions in pairs 1, 2, 3 and 5 in all species, while pair 4 showed signals only in *Colaptes* (Fig 5A and 5B). At interstitial sites, pairs 1 and 3 showed accumulation of (CAC)_10_ and (CAG)_10_ in *Colaptes*, while *M*. *candidus* showed bands with (CAC)_10_, (GC)_15_ and (GAA)_10_ in chromosome 2. For microchromosomes, two different patterns of microsatellite distribution were observed: some accumulated along the total length of the microchromosomes (such as (CA)_15_ and (CGG)_10_), and the rest were observed in the terminal region of the chromosome arms (such as (GAA)_10_, (CG)_15_ and (CAT)_10_).

Interstitial hybridization signals were observed in the long arms of the Z chromosomes. Different patterns of distribution of (GAA)_10_, (GAG)_10_ and (GA)_15_ sequences were found at the three species analyzed, showing three different bands along this chromosome: (GAG)_10_ and (GA)_15_ were found in three bands in *Colaptes*, while in *M*. *candidus* they formed four distinct bands in the Z chromosome. And (CGG)_10_, (CAT)_10_ and (CA)_15_ were found only in *M*. *candidus*, in the terminal region of the Z chromosome (Fig 5A and 5B).

## Discussion

Birds belonging to the Picidae family showed an interesting chromosomal variation, with 2n ranging from 64 to 108 (Table 4). This, along with their large Z chromosome [11], makes them an interesting group for cytogenetic analyses, especially for studies involving the distribution of repetitive sequences, usually associated with morphological differentiation of sex chromosomes [9].

**Table 4:** Diploid number of Picidae species.

Species	2n	References
*Dryocopus martuis*	88	[11]
*Colaptes campestris*	84	This paper
*Colaptes melanochloros*	84	This paper
*Colaptes auratus*	90	[11]
*Picus canus*	92	[13]
*Picus viridis*	94	[12]
*Dinopium benghalense*	92	[10]
*Melanerpes candidus*	64	This paper
*Sphyrapicus varius*	92	[11]
*Dendrocopos minor*	108	[11]
*Dendrocopos major*	108	[11]
*Dendrocopos hyperythrus*	92	[13]
*Dendrocopos kizuki*	90	[13]
*Dendrocopos leucotos*	92	[13]
*Picoides mahrattensis*	84	[10]
*Picoides villosus*	92	[11]
*Picoides pubescens*	92	[11]
*Jynx torquilla*	90	[13]

The species are ordered from the most derived to most basal, following the phylogeny proposed by Benz et al. [27].

Our data suggest that *C*. *campestris* and *C*. *melanochloros* have similar karyotypes in both, morphology and number of chromosomes, with 2n = 84 each. This same 2n has also been found in another species of this family, *Picoides mahrattensis*, although the only other species from the same genus, *Colaptes auratus*, showed 2n = 90 [11]. Considering molecular phylogeny, it is indicated that *C*. *auratus* is more basal than both, *C*. *campestris* and *C*. *melanochloros* [28], thus, taking into account other 2n in the Picidae family, our findings indicate that the increase or decrease of chromosomes in this family occurs randomly, without a phylogenetic tendency [27].

Although in birds, as in other groups, the occurrence of ITS in the centromeric region may represent the accumulation of repetitive sequences, coincidently similar to telomeres [29], it may also represent evidence of chromosomal fusions, as already documented in other bird species [30]. If this is the case in *Colaptes*, and considering that *C*. *auratus* (2n = 90) is placed in a more basal position in relation to *C*. *campestris* and *C*. *melanochloros*, both with 2n = 84, it can be argued that ITS found in the centromeric region of submetacentric pairs 1, 2 and 3 in species with 2n = 84 confirm the occurrence of three centric fusions, which would decrease the hypothetical basal 2n from 90 to 84. However, we also need the occurrence of a pericentric inversion to explain the difference in the number of arms (92 in *C*. *auratus* to 90 in *C*. *campestris* and *C*. *melanochoros*), but all of these rearrangements can be confirmed only by comparative chromosome painting.

Despite their phylogenetic position, the species analyzed in this study retained the plesiomorphic character of showing 18S rDNA clusters in only one microchromosome pair, as it is in the majority of the bird species analyzed so far, including some basal groups such as Paleognathas *Rhea americana*, *Crypturellus tataupa*, *Tinamus solitarius* and *Pterocnemia pennata* [20, 31, 32]. Interestingly, microsatellite sequence (CGG)_10_ was found in chromosomes possessing the secondary constriction, bearers of 18S rDNA clusters. A similar result was found in the fish *Triportheus trifurcatus* (Characiformes, Characidae), where this sequence exist in the W chromosome, which also bears 18S rDNA [9].

Microsatellite sequences were found in both, macrochromosomes, including the Z and in microchromosomes, with some differences between species and in each of the sequences. Despite the existence of interstitial blocks of repetitive DNA, accumulations of microsatellites were also found in centromeric and telomeric regions of the chromosomes.

Repetitive sequences play an important role in the differentiation of sex chromosomes in different groups, including birds, despite the small amount of these classes of DNA in their genome. For instance, it was found that the sequence (GAG)_10_ is accumulated in the W chromosome of *Gallus gallus* [19]. However, none of the microsatellite sequences used in this study hybridized in the W chromosome of *Colaptes*. Instead, some of these sequences were found accumulated in both Z chromosome arms which could explain the fact that it is the largest chromosome in these species. It represents an unusual example of the Z accumulating more repetitive DNAs than the W chromosome. Similar results have been found in the fish *Hoplias malabaricus*, in which the X chromosome was the preferred site for repetitive DNA accumulation in comparison with the Y [33]. Thus, taking into account that the suppression of recombination between the sex chromosome pair is a prerequisite during the evolution of sex chromosomes and that the accumulation of repetitive sequences usually occurs in non- recombining regions, it is possible to track a close relationship between accumulation of different kinds of microsatellite motifs and the physical differentiation of these chromosomes. Probably this is also the situation for other Picidae species, where the Z chromosome is the largest element of the karyotype.

Overall, it may be said that our FISH analysis showed that several microsatellite sequences are found amplified on the Z chromosome of three species belonging to the Picidae family. This may explain the fact that the Z is the largest element of the karyotype, and that their genome contains the highest number of repetitive sequences compared to other groups of birds. Interestingly, none of the sequences were found accumulated on the W chromosome, although they play an important role in the differentiation of sex chromosomes, and are usually found amplified on Y/W chromosomes. These results suggest that, despite the common origin proposed for the ZW sex system in birds, these chromosomes followed different evolutionary trajectories in each species, indicating a high plasticity for sex chromosome differentiation in this group. Amplifications of microsatellite motifs were also found in macrochromosomes and microchromosomes. However, considering the lack of information concerning the genomic distribution of these sequences, it is not yet possible to make a comparison with other birds.

This work is the first step towards clarifying the role of satellites and microsatellite sequences in the differentiation of sex chromosomes. Future studies involving other groups of birds are needed to increase our knowledge in processes of evolution and differentiation of these chromosomes.
